# Phenotypic and genetic variation of *Triatoma costalimai* (Hemiptera: Reduviidae)

**DOI:** 10.1590/0037-8682-0028-2020

**Published:** 2020-12-21

**Authors:** Daniel Pagotto Vendrami, Walter Ceretti-Junior, Marcos Takashi Obara, Vagner José Mendonça, Eduardo Oyama Lins Fonseca, Antonio Ralph Medeiros-Sousa, Mauro Toledo Marrelli, Rodrigo Gurgel-Gonçalves

**Affiliations:** 1 Universidade de São Paulo, Instituto de Medicina Tropical de São Paulo, São Paulo, SP, Brasil.; 2 Universidade de São Paulo, Faculdade de Saúde Pública, Departamento de Epidemiologia, São Paulo, SP, Brasil.; 3 Universidade de Brasília, Faculdade de Ceilândia, Brasília, DF, Brasil.; 4 Universidade Federal do Piauí, Departamento de Parasitologia e Microbiologia, Teresina, PI, Brasil.; 5 Centro Universitário Senai-Cimatec, Technology Center, Salvador, BA, Brasil.; 6 Universidade de Brasília, Faculdade de Medicina, Laboratório de Parasitologia Médica e Biologia de Vetores, Brasília, DF, Brasil.

**Keywords:** Triatoma costalimai, 16S mtDNA, Connexivum color patterns, Wing morphometry, Chagas disease

## Abstract

**INTRODUCTION:**

We aimed to study intraspecific variation in *Triatoma costalimai*, a potential vector of Chagas disease present in Brazil and Bolivia.

**METHODS:**

We analyzed phenotypic (connexivum color patterns, wing morphometrics) and genetic variation (16S mtDNA) of three Brazilian *T. costalimai* populations. We compared 16S sequences with those of putative Bolivian *T. costalimai* and its sister species, *T. jatai*.

**RESULTS:**

Brazilian populations had different connexivum color patterns and forewing shapes. A 16S mtDNA haplotype network showed a clear separation of Brazilian *T. costalimai* from both *T. jatai* and Bolivian *T. costalimai*.

**CONCLUSIONS:**

We report considerable variability in *T. costalimai* populations.

There are approximately 150 known triatomine species^1^. Almost half of these species are found in Brazil, with at least 25 species being native to the Cerrado savanna^2^. *Triatoma costalimai* Verano & Galvão (1958) and *Triatoma jatai* Gonçalves, Teves-Neves, Santos-Mallet, Carbajal-de-la-Fuente and Lopes (2013) are rock-dwelling species apparently endemic to the Brazilian savanna^3^ ^,^ ^4^. *Triatoma costalimai* has been reported in the states of Bahia, Goiás, Minas Gerais, and Tocantins^2^ ^,^ ^5^. In the wild, *T. costalimai* occupies limestone outcrops in association with rodents, birds, and squamate reptiles; however, the species also invades and colonizes houses and peri-domestic structures in Goiás and Tocantins^6^. *Trypanosoma cruzi* infection in *T. costalimai* may vary from 13.7% (*n* = 839) in specimens caught in and around houses^6^ to 64.2% (*n* = 53) in specimens caught in periurban rock outcroppings^7^. These data indicate that *T. costalimai* is a potential vector of Chagas disease. The closely related and morphologically similar *T. jatai* has so far only been reported in southeastern Tocantins, where wild populations are found in rock outcrops and house-invading specimens have been reported sporadically. *Triatoma jatai* differs from *T. costalimai* in color, wing size, and external structures of the male genitalia^4^. The analysis of their two mtDNA gene fragments (16S and COI) supports the hypothesis that *T. jatai* is a sister species of *T. costalimai* ^8^.

*Triatoma costalimai* has also been reported in Cochabamba, Bolivia^9^. In addition, we collected *T. costalimai* specimens from the Brazilian states of Goiás and Bahia that had different connexivum color patterns^10^. Here, we aimed to investigate intraspecific variation in *T. costalimai* by analyzing phenotypic (connexivum color patterns, wing morphometrics) and genetic data (mtDNA 16S sequences) of populations from Brazil (Bahia plus central and northeast Goiás) and Bolivia.

We sampled *T. costalimai* between 2014 and 2016 in three municipalities: Carmo do Rio Verde, Posse, and São Desidério (see geographical information in the  Supplementary Material, Table 1). In each municipality, we collected triatomines manually using forceps over three consecutive days. In São Desidério, we complemented daytime collections with two collections three hours after dusk to increase the number of specimens. The presence of *T. cruzi*-infected specimens of *T. costalimai* was observed in São Desidério (DPV, personal communication). We defined wild environments as rocky outcrops located more than 300 meters from the nearest human dwelling. We identified triatomines according to Lent & Wygodzinsky^10^ and Gonçalves et al*.* ^4^

Right forewings were mounted using Canada Balsam between microscope slides and cover slips and photographed at 8x magnification with a Leica M205C confocal system. The number of specimens used is shown in the  Supplementary Material. We used tpsDig to digitize nine anatomical landmarks (1, 2, 3, 5, 6, 7, 9, 10, and 13 of Schachter-Broide et al.^11^). Shape and size variables were computed using the tpsRelw version 1.18. We used analysis of variance (ANOVA) of centroid size (CS) to compare forewing size across populations. Forewing shape variation was investigated by canonical variate analysis using the MorphoJ software version 2.0. We assessed the influence of size on wing shape (allometry) by multiple regression, using Procrustes coordinates as the dependent variable and CS as the independent variable.

We extracted DNA from the abdominal tissue of each specimen using the Qiagen DNeasy Blood and Tissue Kit (Qiagen, Crawley, United Kingdom), following the manufacturer’s protocol. Two adult *T. costalimai* specimens from the municipality of Mambaí, state of Goiás^5^, were included to increase the number of sequences analyzed. DNA was amplified using polymerase chain reaction with the 16S primers described by Lyman et al*.* ^12^. Reactions were carried out in a MyCycler thermal cycler (Bio-Rad, Hercules, CA, USA) with 1 μL of template DNA, 4 μL of 5× FIREpol Master mix, 1 μL of primers (10 mM), and 13 μL of ultrapure water. Sequencing reactions (forward and reverse) were carried out with ABI Prism dGTP Big Dye Terminator v3.1 kits (Applied Biosystems, Foster City, CA, USA) and sequenced on a Hitachi/ABI PRISM 3100 Genetic Analyzer/DNA Sequencer at Rede Premium, Faculty of Medicine, University of São Paulo, Brazil. We analyzed the sequences in Chromas version 1.45. When more than two sequences were obtained from a given sampled site, we used DNAsp version 5 to determine the number of haplotypes present in specimens from the site. Genetic relationships between haplotypes were inferred using a parsimonious median-joining network with the software Network 5.0.0.3. We assembled a distance matrix with these sequences using the Kimura 2-parameter model to assess the genetic distance between sequences. We generated the matrix in MEGA 6 and used triatomine sequences from GenBank for the analysis, as shown in the supplemental material. Reference specimens were deposited in the Entomological Collection of the Faculty of Public Health, University of São Paulo, under the following registration numbers: specimens from Posse, northeastern Goiás, E15668 to E15671; specimens from Carmo do Rio Verde, central Goiás, E15672, and E15673; and specimens from São Desidério, Bahia, E15674 to E15677.

We found that specimens from Carmo do Rio Verde (central Goiás) have a typical connexivum color pattern that is similar to that described in Lent and Wygodzinsky^10^(see Figure 56, p. 217), with orange-red markings of variable width extending along the outer connexivum border and the dark, inner portion of the connexivum advancing towards the external border along the intersegmental sutures. However, specimens from São Desidério (Bahia) and Posse (northeastern Goiás) have continuous orange-red markings along the outer edge of the connexivum (Figure 1A), a pattern that resembles the one depicted in Figure 58 (p. 220) of Lent and Wygodzinsky^10^.

The mean forewing CS of the Carmo do Rio Verde population (2.67 ± 1.26 mm, SD) was smaller than that of specimens from São Desidério (2.83 ± 1.76 mm) and Posse (2.87 ± 1.33 mm). ANOVA showed a significant difference in wing size (F = 3.482, df = 2, *p* = 0.04514). Factorial maps of forewing shape variation revealed differences among populations; CV1 distinguished specimens from Carmo do Rio Verde from those caught in São Desidério and Posse (Figure 1B). Regression analysis between shape and size was not statistically significant that revealed absence of allometric effect.

**FIGURE 1:**
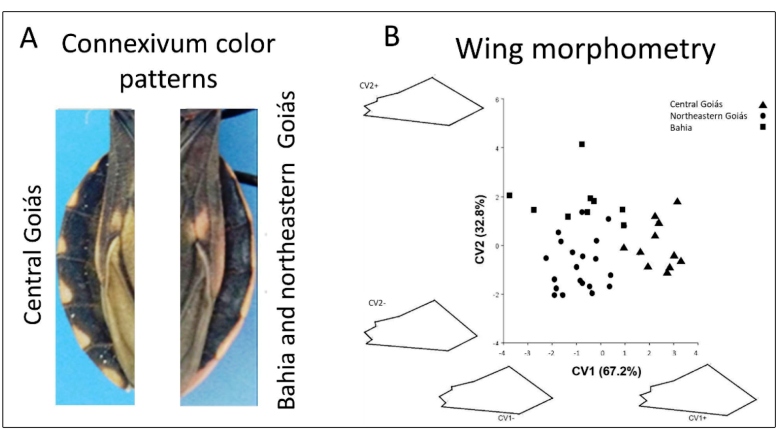
A. Connexivum color patterns found in *Triatoma costalimai* specimens. B. Scatterplot of the first two canonical variate scores for wing shape variation in *Triatoma costalimai* populations. The percentage contribution of each component to the total shape variation is shown in parentheses on the axes. The drawings show the changes in shape at the extremities of the axes.

Thirty-four 16S sequences of 403 bp were obtained: 24 from Carmo do Rio Verde specimens (two haplotypes: CRV1 and CRV2), six from São Desidério specimens (two haplotypes: SD1 and SD2), two from Mambaí (two haplotypes: Mambaí 1 and 2), and two from Posse (one haplotype: P9/P10). A median-joining network with 16S sequences of Brazilian and Bolivian *T. costalimai* and that from *T. jatai* showed clear differences among these groups (Figure 2). The genetic distance between *T. costalimai* sequences from Brazil (Table 1) varied from 0.002 to 0.023; however, the distance between the sequences and the putative *T. costalimai* sequence from Bolivia was greater (0.028 to 0.041). Moreover, the distance between *T. costalimai* and *T. jatai* from Brazil varied from 0.023 to 0.031.

**FIGURE 2:**
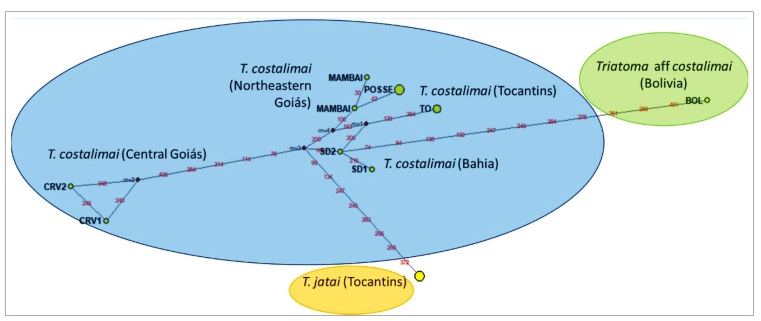
Median-joining network of haplotypes based on *T. costalimai* 16S sequences. Red dots are median vectors, a possible ancestral sequence. CRV1 and CRV2: *T. costalimai* from Carmo do Rio Verde. SD1 and SD2: *T. costalimai* from São Desidério; Mambai: *T. costalimai* from Mambaí; Posse: *T. costalimai* from Posse; TO: *T. costalimai* from Tocantins; Bol: *Triatoma aff. costalimai* from Bolivia; T. jatai: *Triatoma jatai* from Tocantins.

**TABLE 1:** Genetic distance matrix for 16S mtDNA sequences of *Triatoma costalimai*, *T. jatai*, and the Bolivian specimen identified as *T. costalimai*. Analyses were conducted using the Kimura 2-parameter model.

	Species	1	2	3	4	5	6	7	8	9	10	11	12	13	14	15
1	*Triatoma jatai* KT601153.1															
2	*T. jatai* 05 KT601154.1	0.000														
3	*T. jatai* 16 KT601155.1	0.000	0.000													
4	*T. costalimai* TO KT601152.1	0.031	0.031	0.031												
5	*T. costalimai* TO2 KT601151.1	0.031	0.031	0.031	0.000											
6	*T. costalimai* CRV 1 MH538284	0.033	0.033	0.033	0.023	0.023										
7	*T. costalimai* CRV 2 MH538285	0.031	0.031	0.031	0.023	0.023	0.002									
8	*T. costalimai* Mambai 1 MH538286	0.025	0.025	0.025	0.010	0.010	0.020	0.020								
9	*T. costalimai* Mambai 2 MH538287	0.028	0.028	0.028	0.013	0.013	0.023	0.023	0.002							
10	*T. costalimai* Posse KC248997.1	0.028	0.028	0.028	0.013	0.013	0.023	0.023	0.002	0.005						
11	*T. costalimai* Posse 10 MH538288	0.028	0.028	0.028	0.013	0.013	0.023	0.023	0.002	0.005	0.000					
12	*T. costalimai* Posse 9 MH538289	0.028	0.028	0.028	0.013	0.013	0.023	0.023	0.002	0.005	0.000	0.000				
13	*T. costalimai* SD 1 MH538290	0.025	0.025	0.025	0.010	0.010	0.020	0.020	0.010	0.013	0.013	0.013	0.013			
14	*T. costalimai* SD 2 MH538291	0.023	0.023	0.023	0.008	0.008	0.018	0.018	0.008	0.010	0.010	0.010	0.010	0.002		
15	*T.* aff *costalimai* Bolivia KC248998.1	0.041	0.041	0.041	0.033	0.033	0.038	0.036	0.036	0.038	0.038	0.038	0.038	0.030	0.028	

*Triatoma costalimai* showed considerable phenotypic and genetic variation in Brazil*.* A 16S mtDNA haplotype network showed a clear separation of Brazilian *T. costalimai* from both *T. jatai* and Bolivian specimens identified as *T. costalimai*. The connexival segments of *T. costalimai* from central Goiás have orange-red markings of variable widths extending along the outer connexival border, and the pattern is similar to that described by Lent & Wygodzinsky (Figure 56; p. 217)^10^ and Verano & Galvão^3^. However, specimens from Bahia and Northeastern Goiás have continuous markings along the outer edge of the connexival border (Figure 58; p. 220 of Lent & Wygodzinsky^10^)*.* Moreover, these patterns are different from the connexivum of *T. jatai*, which has yellow spots above the intersegmental suture^4^. Triatomines have high morphological plasticity and closely related species develop rapid morphological variations as they adapt to different environments^13^. We suggest that the connexivum color patterns are related to environmental conditions where the triatomines were collected from, and this has been previously observed in *T. patagonica* ^14^. Wing morphometry also revealed differences within *T. costalimai* populations and between *T. jatai* and *T. costalimai* ^4^. The analyses of wing traits may discriminate sibling taxa and reveal fine-scale spatial structuring among populations of a single species^11^. 

The phenotypic plasticity of Triatominae sometimes leads to misidentification of genetically distinct convergent species particularly through the qualitative evaluation of chromatic characters^15^. These mistakes could be consequences of the incorrect and exclusive use of the dichotomous keys of Lent & Wygodzinsky^10^ that disregard the ecological and geographic characteristics of the specimens especially when dealing with phenotypic variation or cryptic species. This seems to be the case for the specimens morphologically similar to *T. costalimai* collected in Bolivia. Incorrect identification might have both systematic and epidemiological implications as taxonomic uncertainties could generate misleading occurrence records that could result in biogeographic inference mistakes^15^.

Although there is no consensus regarding the similarity or difference values of different genetic markers to define a new species of Triatominae, our study revealed a clear genetic distance of 0.041 between the Bolivian and the Brazilian *T. costalimai* based on the analysis of 16S sequences. Analysis by Teves et al.^8^ revealed a distance of 0.025 between *T. jatai* and *T. costalimai* collected in Tocantins based on the analysis of 16S sequences. We report considerable variability in Brazilian *T. costalimai* populations. Moreover, we found substantial mtDNA divergence between the bona fide Brazilian *T. costalimai* and the Bolivian specimen identified as *T. costalimai*. Further studies and the inclusion of new populations are necessary to suggest a new *Triatoma* species from Bolivia. 
