# Supplementary material for: Phenotypic and genetic variation of Triatoma costalimai (Hemiptera: Reduviidae)
Source: Rev Soc Bras Med Trop. 2020 Dec 21;54:e00282020. doi: 10.1590/0037-8682-0028-2020 (PMC7747808; doi:10.1590/0037-8682-0028-2020)
Supplement: Supplementary file 1 [file 1678-9849-rsbmt-54-e00282020-suppl1.pdf]

TABLE 1: Number of *Triatoma costalimai* specimens used in the study sorted by municipalities and methods.

**Municipalities**	**Methods**				
	**Geometrics Morphometrics**		**Molecular Analysis**		
	Habitat	Adults	Habitat	Adults	Nymphs
Posse (Northeastern Goiás)					
	Rocks	15	Rocks	1	
	F1*	5	Chicken coop	1	
Carmo do Rio Verde (Central Goiás)					
	Rocks	5	Rocks		1
		6	Rocks		10
			Rocks		8
			Rocks		16
São Desidério (Bahia)					
	Rocks	9	Rocks	1	36

*F1 generation.

TABLE 2: Municipalities, locations, and habitats where *Triatoma costalimai* specimens were collected in Brazil.

**Municipality**	**Location**	**Habitat**	**GPS***	**Date**
Posse (Northeastern Goiás)				
	Jatobá de Baixo	Wild area	S 14° 10' 34.4" W 46° 22' 31.8"	Dec/2014
	Jatobá de Baixo	Chicken coop	S 14º 11' 40.5" W 46° 22' 41.7"	Dec/2014
Carmo do Rio Verde (Central Goiás)				
	Pedra Solta	Wild area	S 15° 29' 25.9" W 49° 51' 06.4"	Jun/2016
	Pedra Solta	Wild area	S 15º 28' 39.6" W 49º51'16.9"	Jun/2016
	Morro Alegre	Wild area	S 15º 27' 52.8" W 49º50'20.9"	Jun/2016
	Pedra Solta 1	Wild area	S 15º 30' 05.0" W 49º 49' 46.2"	Jun/2016
São Desidério (Bahia)				
	Baixo dos Coqueiros	Wild area	S 12°29'55.6" W 044°54'55.2"	Aug/2016

***** For the acquisition of the geographic coordinates, we utilized eTrax H (Garmin) GPS. The datum utilized was WGS84.

TABLE 3: GenBank accession numbers, habitats, and origin of triatomine species analyzed.

Species	16S	Habitat	Origin
Triatoma jatai	KT601153.1, KT601155.1, KT601154.1	Rocks	Paranã, Tocantins
Triatoma costalimai	KT601152.1, KT601151.1	Rocks	Aurora do Tocantins, Tocantins
	KC248997	Rocks	Posse, Goiás
	KC248998.1	?	Cochabamba, Bolivia
	**MH538284**	Rocks	Carmo do Rio Verde, Goiás
	**MH538285**	Rocks	Carmo do Rio Verde, Goiás
	**MH538286**	Rocks	Mambaí, Goiás
	**MH538287**	Rocks	Mambaí, Goiás
	**MH538288**	Rocks	Posse, Goiás
	**MH538289**	Rocks	Posse, Goiás
	**MH538290**	Rocks	São Desidério, Bahia
	**MH538291**	Rocks	São Desidério, Bahia

Sequences obtained in this study are highlighted in bold.
